# Tumor integrin targeted theranostic iron oxide nanoparticles for delivery of caffeic acid phenethyl ester: preparation, characterization, and anti-myeloma activities

**DOI:** 10.3389/fphar.2024.1325196

**Published:** 2024-03-06

**Authors:** Barkley Smith, Yuancheng Li, Travis Fields, Michael Tucker, Anna Staskiewicz, Erica Wong, Handong Ma, Hui Mao, Xinyu Wang

**Affiliations:** ^1^ Department of Pharmaceutical Sciences, School of Pharmacy, Philadelphia College of Osteopathic Medicine–Georgia Campus, Suwanee, GA, United States; ^2^ 5M Biomed, Limited Liability Company, Atlanta, GA, United States; ^3^ Department of Radiology and Imaging Sciences, Emory University, Atlanta, GA, United States; ^4^ Division of Research, Philadelphia College of Osteopathic Medicine–Georgia Campus, Suwanee, GA, United States

**Keywords:** multiple myeloma, CAPE, RGD, IONP, apoptosis, tumor microenvironment

## Abstract

Multiple myeloma (MM) is characterized by the accumulation of malignant plasma cells preferentially in the bone marrow. Currently, emerging chemotherapy drugs with improved biosafety profiles, such as immunomodulatory agents and protease inhibitors, have been used in clinics to treat MM in both initial therapy or maintenance therapy post autologous hematopoietic stem cell transplantation (ASCT). We previously discovered that caffeic acid phenethyl ester (CAPE), a water-insoluble natural compound, inhibited the growth of MM cells by inducing oxidative stress. As part of our continuous effort to pursue a less toxic yet more effective therapeutic approach for MM, the objective of this study is to investigate the potential of CAPE for *in vivo* applications by using magnetic resonance imaging (MRI)-capable superparamagnetic iron oxide nanoparticles (IONP) as carriers. Cyclo (Arg-Gly-Asp-D-Phe-Cys) (RGD) is conjugated to IONP (RGD-IONP/CAPE) to target the overexpressed α_v_β_3_ integrin on MM cells for receptor-mediated internalization and intracellular delivery of CAPE. A stable loading of CAPE on IONP can be achieved with a loading efficiency of 48.7% ± 3.3% (wt%). The drug-release studies indicate RGD-IONP/CAPE is stable at physiological (pH 7.4) and basic pH (pH 9.5) and subject to release of CAPE at acidic pH (pH 5.5) mimicking the tumor and lysosomal condition. RGD-IONP/CAPE causes cytotoxicity specific to human MM RPMI8226, U266, and NCI-H929 cells, but not to normal peripheral blood mononuclear cells (PBMCs), with IC50s of 7.97 ± 1.39, 16.75 ± 1.62, and 24.38 ± 1.71 μM after 72-h treatment, respectively. Apoptosis assays indicate RGD-IONP/CAPE induces apoptosis of RPMI8226 cells through a caspase-9 mediated intrinsic pathway, the same as applying CAPE alone. The apoptogenic effect of RGD-IONP/CAPE was also confirmed on the RPMI8226 cells co-cultured with human bone marrow stromal cells HS-5 in a Transwell model to mimic the MM microenvironment in the bone marrow. In conclusion, we demonstrate that water-insoluble CAPE can be loaded to RGD-IONP to greatly improve the biocompatibility and significantly inhibit the growth of MM cells *in vitro* through the induction of apoptosis. This study paves the way for investigating the MRI-trackable delivery of CAPE for MM treatment in animal models in the future.

## Graphical Abstract

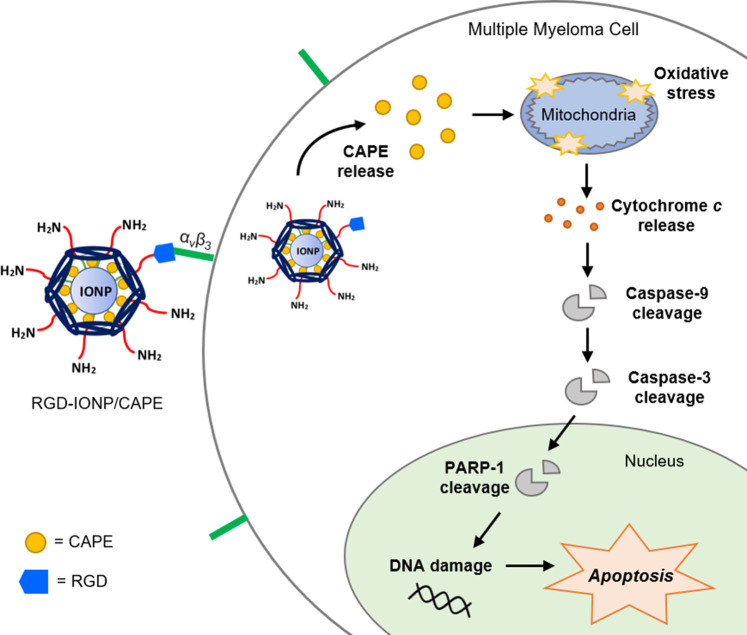

## Introduction

Multiple myeloma (MM) is a type of hematological cancer that affects plasma cells in the bone marrow. Currently, MM has no cure, with a 5-year relative survival rate of 55% (Daniele et al., 2022). It is estimated that there will be 35,730 new cases and 12,590 deaths in the United States in 2023 (Siegel et al., 2023). In MM, a mass of malignant plasma cells forms within the bone marrow, crowding out the normal plasma cells and preventing them from functioning normally, such as producing antibodies for the immune system (Kazandjian, 2016). Although the underlying disease biology remains not fully elucidated (Kumar et al., 2017), an increasing body of evidence has suggested that MM is not a single disease but rather a collection of diseases with common clinical manifestations (Kyle et al., 2007). Treatment regimens for MM are closely related to the transplant eligibility of patients (Dimopoulos et al., 2021; Rajkumar, 2022), as some chemotherapy drugs, such as melphalan, may cause hematological toxicity affecting the subsequent autologous hematopoietic stem cell transplantation (ASCT) (Knudsen et al., 1999). The approaches for treating MM have been improved over the years (Rajkumar, 2019; Rajkumar, 2022). The combination of proteasome inhibitors (e.g., bortezomib) and immunomodulatory agents (e.g., lenalidomide) is currently one of the most effective initial therapies for transplant-eligible patients of MM. The proteasome inhibitors and immunomodulatory agents can also be used in combination with melphalan and prednisone for those ineligible for ASCT (Palumbo et al., 2006; Facon et al., 2007; San Miguel et al., 2008).

To discover more effective yet safer chemotherapy approaches for MM, we have been investigating natural compounds that exhibit reduced side effects compared to current chemotherapies. In particular, caffeic acid phenethyl ester (CAPE), a water-insoluble phenolic natural compound identified in honeybee hive propolis, has been used in folk medicine for centuries demonstrating a plethora of beneficial biological properties, including anti-microbial (Niu et al., 2020), anti-inflammatory (Semis et al., 2021), antioxidant (Gocer and Gulcin, 2011), anti-viral and protective effects against ischemia–reperfusion-induced injury in multiple tissues (Tolba et al., 2016). More importantly, CAPE has shown anti-cancer effects *in vitro* for human pancreatic cells, human colon cancer cells, and C6 glioma cells (Murtaza et al., 2014; Lv et al., 2021). Previously we discovered that CAPE inhibited the growth of MM cells by inducing apoptosis through oxidative stress whilst maintaining non-toxic for normal human blood cells (Marin et al., 2019). In addition, an *in vitro* study has shown that CAPE and its analogues significantly reduced the level of interferon regulatory factor-4 (IRF-4) in MM cells, a crucial transcription factor controlling the defenestration of plasma cells (Murugesan et al., 2020). The mechanism of action for CAPE was linked to the downregulation of specificity protein 1 (Sp-1) and its downstream IKZF1-IRF4-MYC axis. Moreover, CAPE has shown inhibitory effects on the drug efflux transporter P-glycoprotein (Nabekura et al., 2015), which is responsible to a great extent for the chemo-resistance in various cancer cells including MM (Cree and Charlton, 2017; Uckun, 2022). All the previously reported work suggested the effectiveness of CAPE in treating MM cells might be from mechanisms that are different from those of currently used chemotherapies. However, the *in vivo* applications of CAPE as therapeutics for cancer treatment are significantly hindered by its insolubility in water, rapid degradation *in vivo*, short half-life, and the lack of effective delivery approach to cancer cells (Celli et al., 2007; Wang et al., 2007; Wang et al., 2009; Gao and Hu, 2010; Khushnud and Mousa, 2013).

Nanoparticles (NP) are effective delivery platforms for therapeutic agents, particularly the potent yet hydrophobic ones, to improve the pharmacokinetics (PK), pharmacodynamics (PD), delivery efficiencies to diseased sites while reducing systemic cytotoxicity (Singh et al., 2018; Mitchell et al., 2021), and even therapeutic efficacies (Watkins et al., 2015). Although constructing polymeric NPs to carry CAPE for *in vivo* uses have been investigated (Lee et al., 2015; Wang et al., 2020b; Nasrullah, 2022), application of IONP as drug carriers for CAPE is rarely reported, not mentioning leveraging the superb magnetic resonance imaging (MRI) contrast enhancement of superparamagnetic IONP for theranostic applications *in vivo* (Huang et al., 2016; Vangijzegem et al., 2019). In this *in vitro* study, we investigated the approach of encapsulating CAPE onto iron oxide nanoparticles (IONP) for improved bioavailability and cytotoxicity specific to human MM cells, and validated its potential for MRI-guided delivery of CAPE for MM in future animal studies. IONP with a polyethylene glycol-*block*-allyl glycidyl ether (PEG-*b*-AGE) di-block polymer coating were selected as the carriers, given the capabilities in reducing non-specific interactions with bio-molecules and preserving specificity for targeted cells *in vitro* and *in vivo* (Li et al., 2015; Lin et al., 2017; Li et al., 2020; Li et al., 2022). Cyclo (-Arg-Gly-Asp-D-Phe-Cys) (RGD) was conjugated to IONP as ligands for targeting MM cancer cells through binding of α_v_β_3_ integrin that are overexpressed on the surface of MM cells, and the subsequent integrin-facilitated internalization to deliver CAPE intracellularly (Ria et al., 2002; Ludwig et al., 2021; Egorova and Nikitin, 2022). The RGD-conjugated IONP with CAPE loading (RGD-IONP/CAPE) was characterized for the loading efficiency and release profile of CAPE under different pH conditions, as well as the MRI contrast enhancement. The targeting specificity and apoptogenic effects of RGD-IONP/CAPE for MM cells were assessed. A Transwell (TW) model with co-cultured human MM cells and bone marrow stromal cells, which allows for the signaling between MM and stromal cells (Rogers et al., 2020), was employed to mimic the microenvironment of MM and evaluate RGD-IONP/CAPE with more clinical relevance.

## Materials and methods

### Chemicals and reagents

IONP with core diameter of 10 nm and anti-biofouling PEG-*b*-AGE polymer coating (SKU: NPABW010) was provided by 5M Biomed, LLC (Atlanta, GA, USA). Polyethylene glycol with molecular weight of 550 g/mol (PEG550) was purchased from Sigma-Aldrich (St. Louis, MO, USA). Cyclic peptide cyclo-(RGDfC) and cyclo-(RADfC) were purchased from Peptide International, Inc. (Louisville, KY, USA). CAPE (≥98%) was obtained from Cayman Chemicals (Ann Arbor, MI, USA). Sulfosuccinimidyl 4-(N-maleimidomethyl)cyclohexane-1-carboxylate) (sulfo-SMCC), fluorescein isothiocyanate (FITC), Presto Blue cell viability reagent, fetal bovine serum (FBS), penicillin, and streptomycin were all purchased from Thermo Fisher Scientific (Grand Island, NY, USA). FITC Annexin V Apoptosis Detection Kit was purchased from BD Biosciences (San Jose, CA, USA).

### Encapsulation of CAPE to anti-biofouling IONP

Anti-biofouling water soluble IONP were firstly examined by transmission electron microscopy (TEM, HT7700, Hitachi, Tokyo, Japan) for morphology. The core diameter of IONP was determined by averaging 30 randomly picked IONP in a TEM image. The hydrodynamic diameter was measured by dynamic light scattering on a Zetasizer Nano ZS90 (Malvern Panalytical Inc., Malvern, United Kingdom) with the surface zeta-potential measured on the same instrument. The encapsulation of IONP with CAPE was then performed by adding CAPE (5 mg) to the IONP aqueous solution (1 mg Fe/mL, 5 mL) in a centrifuge tube. The tube was kept inverting on a shaker for 6 h at room temperature before PEG550 (1 mL) was added. The mixture in the tube was kept inverting for additional 24 h, followed by centrifuging at 3,000 rpm for 5 min to remove un-loaded CAPE and large aggregates of IONP. The supernatant was then collected and filtrated using an Amicon^®^ ultra-4 centrifugal filter unit with a cutoff molecular weight of 100k Dalton to remove free PEG550, and the filtrated IONP/CAPE was re-suspended with deionized (DI) water. The centrifugal filtration and re-suspension were repeated three times.

To determine the loading efficiency of CAPE to the IONP (wt%, CAPE/Fe), the Fe concentration of IONP/CAPE was measured using a 1,10-phenanthroline colorimetric assay (Huang et al., 2013) and the CAPE content was quantified by the UV absorbance at 330 nm (Shahin et al., 2022). Briefly, a standard curve of CAPE absorbance with a concentration range from 0 to 0.065 mg/mL was established firstly. The UV absorbance of IONP/CAPE and IONP with the same Fe concentration was then measured at 330 nm on a UV-Vis spectrophotometer (Genesys 50, Thermo Scientific, Waltham, MA, USA), the difference of which was used for CAPE quantification based on the standard curve. The CAPE loading efficiency was defined as:

$$\text{Loading Efficiency}=\left[\text{CAPE}\right]/\left[\text{IONP}\right]\times 100\%$$ (1)

where [CAPE] and [IONP] were the CAPE and Fe concentrations (in mg/mL) of IONP/CAPE in water.

### Functionalization of IONP/CAPE with RGD and FITC

RGD was conjugated to the IONP/CAPE as a targeting ligand for α_v_β_3_ integrin overexpressed by RPMI8226 MM cells. The conjugation of RGD with IONP/CAPE was carried out through the NH_2_- groups on the surface of IONP/CAPE and the -SH group of RGD’s cysteine following previously published protocol (Li et al., 2022). Briefly, the IONP/CAPE was firstly activated with maleimide groups by incubating IONP/CAPE with sulfo-SMCC in PBS for 2 h to allow the coupling between NH_2_- groups on IONP/CAPE and NHS ester of sulfo-SMCC. The conjugation of RGD was then realized *via* a “click” chemistry between -SH and maleimides by incubating SMCC-IONP/CAPE with RGD in PBS for 1 h. Afterwards, the unconjugated sulfo-SMCC and RGD were removed by filtration of the RGD-IONP/CAPE solution using an Amicon^®^ ultra-4 centrifugal filter unit (COMW 100k). The collected RGD-IONP/CAPE was re-suspended in DI water. RGD conjugated on the surface of IONP were quantified using microBCA assay following the manufacturer’s manual. The number of RGD conjugated to each IONP was determined by the molar ratio of RGD content to the concentration of IONP measured by 1,10-phenanthroline colorimetric assay described above with the assumption that the nanoparticle was spherical with a bulk magnetite density of 5.18 g cm^-3^.

**FIGURE 1:**
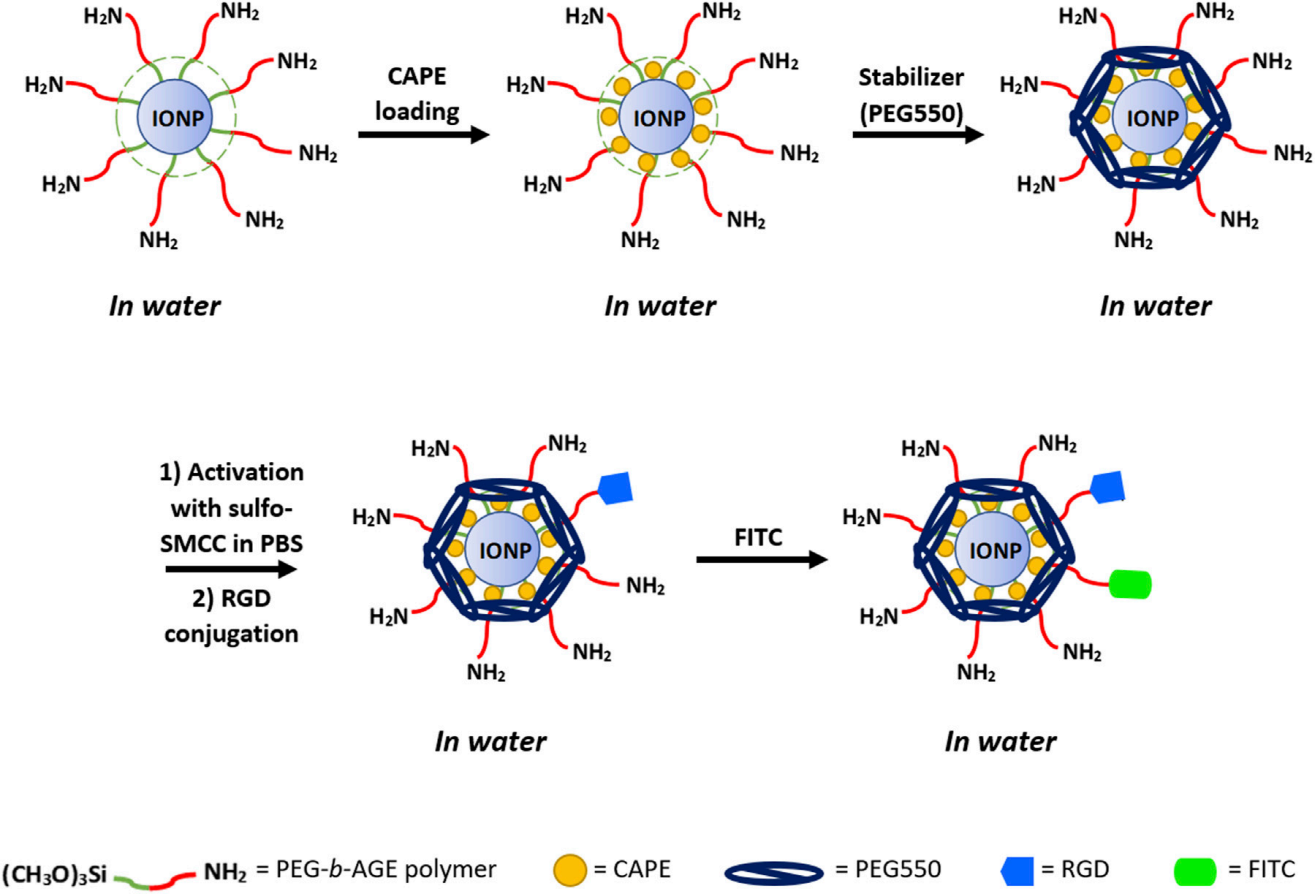
Illustration of the preparation of RGD-IONP/CAPE labeled with FITC fluorescent dye.

To fluorescently label IONP for optical imaging and flow cytometry analysis, fluorescence tagging FITC was conjugated to RGD-IONP/CAPE using the labeling protocol published in the literature (Lin et al., 2017). In a typical procedure, RGD-IONP/CAPE solution (1 mg Fe/mL) was prepared in a carbonate buffer (250 mM, pH 9.5), to which FITC dissolved in dimethyl sulfoxide (DMSO, 10 mg/mL) was added with the molar ratio of FITC/IONP equal to 50:1. The mixture of FITC and RGD-IONP/CAPE were incubated for 1 h to yield the FITC-labeled RGD-IONP/CAPE. The hydrodynamic diameters and zeta-potentials were measured for IONP/CAPE before and after the functionalization of RGD and FITC to validate the conjugations. The preparation of RGD-IONP/CAPE labeled with FITC is illustrated in Figure 1.

### Release of CAPE from IONP/CAPE in different pH

The release profile of IONP/CAPE was investigated by measuring the CAPE contents of IONP/CAPE at 1, 2, 4, 24 and 48 h after exposing to three different pH (i.e., 5.5, 7.4 and 9.5), which resembled the lysosomal, physiological and FITC labeling conditions, respectively. The experiments followed the procedure we established previously (Li et al., 2022). Briefly, IONP/CAPE solution in water (1 mg Fe/mL) was added to acetate buffer (0.1 M, pH 5.5), PBS (1X, pH 7.4) and carbonate buffer (0.5 M, pH 9.5) in 2 mL centrifuge tubes, respectively, to reach the final volume of 1.8 mL and final Fe concentration of 0.2 mg/mL. The solutions were incubated at room temperature. At each time point, a portion of solution (300 µL) was taken out and centrifuged at 3,000 rpm for 10 min to allow the released CAPE to precipitate. The resultant supernatant was then taken to measure the un-released CAPE contents in IONP/CAPE using a UV-Vis spectrophotometer as described above. Each measurement was repeated three times.

### MRI of IONP complexes

The MRI contrast enhancement of IONP before and after CAPE encapsulation and surface modification was evaluated by the transverse relaxivity (r_2_) through procedure described previously (Li et al., 2015). MRI was performed on a 3T scanner (Prisma, Simens, Erlangen, Germany) using a head coil. Phantom solutions of IONP and RGD-IONP/CAPE were prepared with the Fe concentrations of 0.03, 0.06, 0.12, and 0.25 mM. A multi-echo spin echo sequence was performed with key parameters as follow: TR = 2,520 ms, 20 TEs starting at 13.1 ms with increments of 13.1 ms. ImageJ (National Institutes of Health, Bethesda, MD, USA) was used for data analysis. Briefly, regions of interest (ROI) were drawn for each sample solution to measure the mean signal intensities at all TEs. The transverse relaxation time (T_2_) was calculated using an exponential curve fitting based on the equation below.

$$SI=Ke^{-TE/T2}$$ (2)

where SI was the averaged signal intensities, and K was the constant generated by the fitting. The r_2_ values (unit mM^-1^·s^-1^) for IONP and RGD-IONP/CAPE were then calculated as the slopes of linear plots between Fe concentrations and 1/T_2_.

### Cell lines and cell culture

Three human MM cell lines (RPMI8226, NCI-H929, and U266), human primary peripheral blood mononuclear cells (PBMCs), human bone marrow stromal cell line HS-5, murine macrophage cell line RAW264.7, culturing medium RPMI 1640, and DMSO were purchased from American Type Culture Collection (ATCC, Manassas, VA, USA). RPMI8226 cells (Cat# CCL-155) were cultured using RPMI 1640 medium supplemented with 10% FBS and 1% penicillin/streptomycin. NCI-H929 cells (Cat# CRL-3580) were maintained in RPMI 1640 medium supplemented with 10% FBS, 1% penicillin/streptomycin, and 0.2% 2-mercaptoethanol. U266 cells (Cat# TIB-196) were cultured using RPMI 1640 medium supplemented with 15% FBS and 1% penicillin/streptomycin. HS-5 stromal cells (Cat# CRL-3611) were cultured with RPMI 1640 medium supplemented with 10% FBS and 1% penicillin/streptomycin. RAW264.7 macrophage cells (Cat# TIB-71) were cultured with DMEM medium supplemented with 10% FBS and 1% penicillin/streptomycin. Human PBMCs (Cat# PCS-800–011) containing a mixture of mononuclear blood cells were first rinsed with Hank’s Balanced Salt Solution without Ca^2+^ or Mg^2+^ supplemented with 10% FBS, and then re-suspended in RPMI 1640 medium supplemented with 10% FBS and 1% penicillin/streptomycin to maintain the viability, following the manufacturer’s procedure from ATCC (https://www.atcc.org/products/pcs-800-011). Three vials of PBMCs from ATCC were used independently as biological replicates to determine the cytotoxicity. For each vial of PBMCs, three technical replicates were performed for the study. All cells were maintained at 37°C in the presence of 5% CO_2_ and 95% air in a humidified incubator. The human MM cell lines, bone marrow stromal cell line HS-5, and murine macrophage cell line RAW264.7 were supplemented with fresh medium every 2–3 days to maintain appropriate cell concentration in the medium according to the manufacturer’s instruction (ATCC).

### *In vitro* cell targeting to RPMI8226 MM cells

The targeting of RGD-IONP/CAPE for the RPMI8226 cells with overexpression of α_v_β_3_ integrin was validated by fluorescence assisted cell sorting (FACS), with RAW264.7 murine macrophage cells presenting low expression of α_v_β_3_ integrin as the negative control. The experiments were carried out following a procedure modified from the one we established previously (Li et al., 2022). Briefly, FITC-labeled IONP/CAPE and RGD-IONP/CAPE were incubated with 10^6^ RPMI8226 MM or RAW264.7 macrophage cells for 3 h at 37°C with the Fe concentration at 0.1 mg/mL. The cells were then centrifuged and re-suspended in PBS to assess the binding of FITC-RGD-IONP/CAPE to the MM cells through the measurement of FITC intensities from cells using a BD FACSymphony A3 flow cytometer (BD Biosciences, San Jose, CA, USA). The fluorescence measurements of FITC tagging (excitation 488 nm, emission 525 nm) were repeated three times for 1×10^5^ cells each time.

### Cell viability assays

We previously determined the cytotoxic effects of CAPE in MM cells. Here, we followed the same procedure (Marin et al., 2019). Briefly, a total of 40,000 viable cells were plated in each well of 48-well plates immediately prior to treatment. MM cells were treated with RGD-IONP/CAPE at various concentrations (5, 10, 20 and 30 µM), IONP/CAPE at 30 μM, CAPE only (30 µM), and RGD-IONP as control. MM cells were incubated with the compounds for 24, 48 and 72 h. Presto Blue reagent was added to measure the viability of MM cells following the manufacturer’s protocol. After 2 hours of incubation at 37°C, the resulting fluorescent intensity was measured at 545/590 nm (excitation/emissions) using a Synergy HT plate reader (BioTek Instruments Inc., Winooski, VM, USA). Each treatment was repeated three times and three independent experiments were conducted to confirm consistency and determine statistical significance. The cytotoxicity data are presented as mean ± SD of three independent replicates.

### Flow cytometry analysis

Apoptosis of RGD-IONP/CAPE treated RPMI8226 cells was examined using an Annexin V-FITC (AV)/7-Amino-Actinomycin (7-AAD) dual staining of cells followed by flow cytometry analysis as previously reported (Staskiewicz et al., 2023). Cultured RPMI8226 cells (1.0 × 10^6^ cells/well) were plated in 6-well plates and treated with RGD-IONP/CAPE at various concentrations (1, 5, 10, 20 and 30 µM), IONP/CAPE at 30 μM, CAPE only (30 µM), and RGD-IONP as control for 24 h. After treatment, cell staining was performed using a FITC Annexin V Apoptosis Detection Kit following the manufacturer’s protocol. Briefly, the treated cells were isolated, washed with PBS, and resuspended in 1 mL of the binding buffer from the kit. Collected cells in 100 µL binding buffer were stained with 5 µL each of FITC Annexin V and 7-AAD fluorescent dyes. After 15 min incubation in the dark at room temperature on a shaker, those stained samples were loaded onto the BD Accuri™ C6 Flow Cytometer (BD Biosciences, San Jose, CA, USA). The samples were run through the flow cytometer at a medium speed to collect 10,000 events. Normal and apoptotic cell populations were gated and quantified using BD Accuri™ C6 software (version 1.0, BD Biosciences). Each treatment was repeated in three independent experiments.

### Western blotting

RPMI8226 ells were treated with cell only control, IONP-RGD control, IONP/CAPE at 30 μM, CAPE only at 30 μM, and 1, 5, 10, 20 and 30 µM of RGD-IONP/CAPE for 24 h. Protein from treated cells was collected using complete 1X RIPA lysis buffer (Thermo-Fischer Scientific Inc., MA, USA). Protein concentration was estimated using the Pierce™ BCA assay kit (Thermo-Fischer Scientific). Equal masses of protein (30 µg) collected from each treatment were loaded and run on 10-well 4%–20% Mini-PROTEAN^®^ TGX™ precast gel (Bio-Rad, Hercules, CA, USA) at 100V for one and a half hours. Protein was then transferred from the gel to an Immuno-Blot PVDF membrane (Bio-Rad, Herculus, CA, USA). After the transfer was completed, the membrane was washed 3 times for 5 min each, respectively, with PBST (1X PBS with 0.1% Tween20). The membrane was then blocked for an hour at room temperature using Li-COR blocking buffer (Li-COR Biosciences, Lincoln, NE, USA). Primary antibodies for caspase-3, caspase-8, caspase-9, PARP-1, and housekeeping GAPDH or β-actin (AbCam, Cambridge, MA, USA) as loading control were diluted in Li-COR blocking buffer at a ratio of 1:1,000 and incubated overnight at 4°C. After incubation, the membrane was washed for three times with PBST for 5 min each time. The blot was then incubated with Li-COR IRDye^®^ secondary antibodies at a ratio of 1:10,000 for 30 min at room temperature. After additional washes for 5 minutes three times, the membrane was imaged with the Li-COR Odyssey CLX imaging system (LI-COR Biosciences - U.S., NE, USA). Protein bands are detected using the 700 and 800 channels. Intensity of target protein bands was quantified using the Image Studio software™ (Li-COR, version 5.2) (Pillai-Kastoori et al., 2020). Each treatment was repeated in three independent experiments.

### TW model

In order to examine the effect of IONP-encapsulated CAPE on MM cells in a condition simulating the bone marrow microenvironment, we adapted a TW model (Perez et al., 2008) that has been applied to examine the inhibitory effects of potential drug candidates on MM cell growth (Rogers et al., 2020). Briefly, human HS-5 stromal cells were plated at 2.5 × 10^5^ cells per well in the basal chambers of a 6 well TW plate (Corning Inc., Life Sciences, Lowell, MA, USA) 24 h before the RPMI8226 cells were plated in the apical chamber. RPMI8226 cells were cultured on the TW inserts (Costar, 0.4-µm mesh; Corning Inc.) at 5 × 10^5^ cells per well. RGD-IONP/CAPE treatment (0, 1, 5, 10, 20 and 30 µM) along with other controls were added to the inserts. After incubating for 24 h, the RPMI8226 cells in the apical chamber were collected and subjected to flow cytometry analysis and Western blotting as described above, respectively, to determine the induction of apoptosis. Each treatment was repeated in three independent experiments.

### Statistical analysis

Statistical analysis was conducted using GraphPad Prism version 8.0.2 (GraphPad Software, San Diego, CA, USA). The half-maximal inhibitory concentration (IC50) was determined using equations of “[inhibitor] vs. response” from the panel of equations “Dose-response curves - Inhibition” under analyses of nonlinear regression. The results in the bar charts were presented as mean ± standard deviation (SD). Differences between and within groups were analyzed using one-way analysis of variance, and, if found significant, the analyses were followed by *post hoc* tests of Tukey (equal variances assumed) or Games-Howell (equal variances not assumed) for inter-group comparisons. A *p*-value ≤0.05 was considered statistically significant.

## Results

### Preparation, functionalization and characterization of CAPE loaded IONP

**FIGURE 2:**
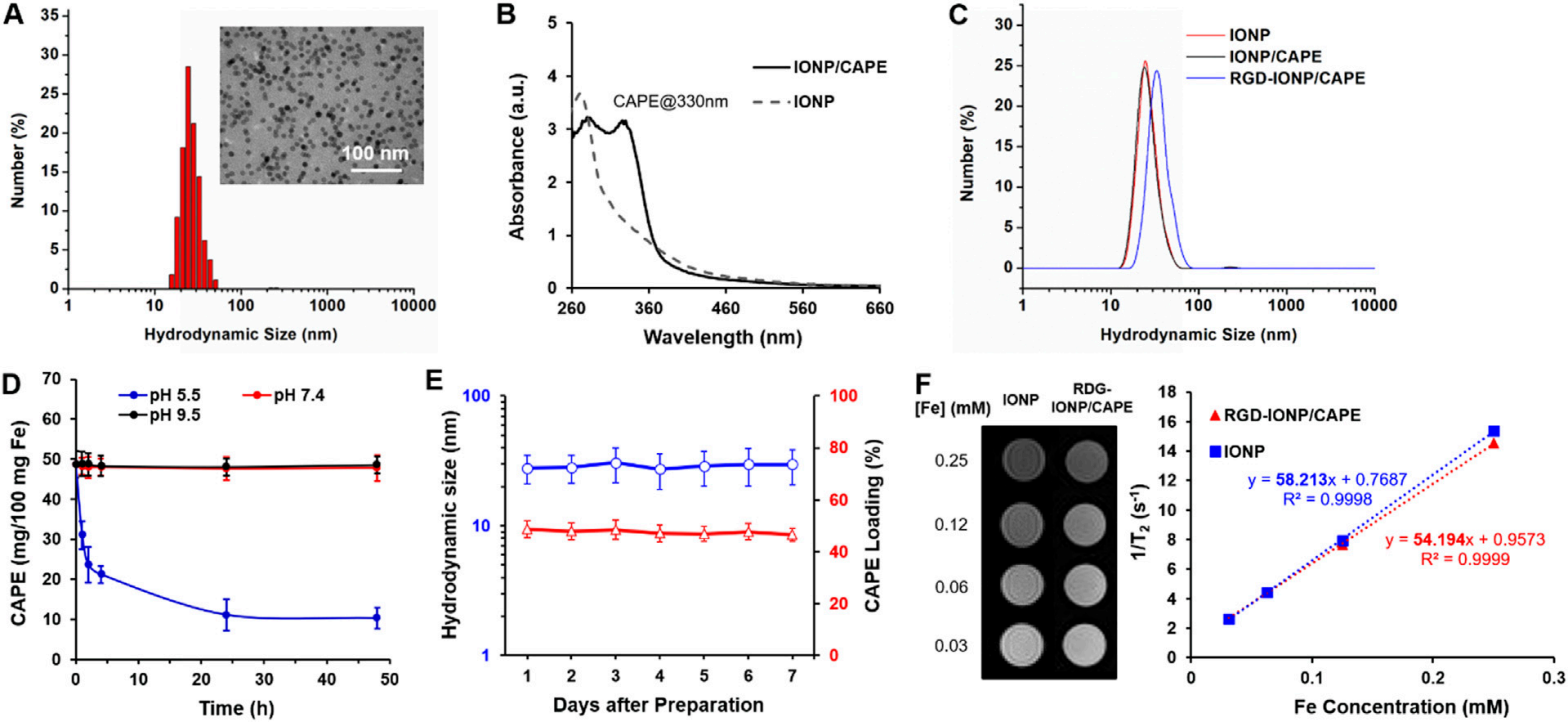
**(A)** Characterization of hydrodynamic size of IONP with 10 nm core diameter (inset: TEM image of IONP). **(B)** UV-Vis spectra of IONP and IONP/CAPE. **(C)** Change of hydrodynamic sizes of IONP after CAPE encapsulation and subsequent RGD conjugation. **(D)** Stability profiles CAPE loading of RGD-IONP/CAPE under acidic (pH 5.5), neutral (pH 7.4) and basic (pH 9.5) conditions. **(E)** Monitoring of the hydrodynamic sizes and CAPE loading of IONP/CAPE for 7 days. **(F)** T_2_-weighted spin echo MR images of IONP and RGD-IONP/CAPE at different Fe concentrations, and comparison of transverse relaxivities of IONP and RGD-IONP/CAPE determined by transverse relaxation rate (R2 or 1/T_2_) at different Fe concentrations (mM).

As shown in the TEM image, the water-soluble, anti-biofouling IONP with PEG-*b*-AGE polymer coating were monodispersed and highly uniform in size with an averaged core diameter of 10.8 ± 0.2 nm (Figure 2A inset). The dynamic light scattering (DLS) measured hydrodynamic diameter (D_H_) of IONP showed an average of 27.9 nm with the polydispersity index (PDI) of 0.12 (Figure 2A), indicating a narrow distribution of IONP size. Like other drug carrying IONP with amphiphilic polymer coating, the anti-biofouling IONP have been shown to encapsulate hydrophobic drugs, such as 7-ethyl-10-hydroxycamptothecin (SN-38) and doxorubicin (Xie et al., 2020; Li et al., 2022; Xue et al., 2022), to achieve targeted deliveries for cancer treatment. As expected, hydrophobic CAPE molecules were encapsulated to the anti-biofouling polymer coated IONP, evidenced by an increased UV absorbance at the characteristic peak of CAPE at 330 nm (Shahin et al., 2022), as shown in Figure 2B. However, the CAPE loading was not stable, exhibiting a release of more than 50% of loaded CAPE at pH 7.4 within 24 h (Supplementary Figure S1). To improve the loading stability for CAPE, we added low molecular weight polyethylene glycol (PEG550) during drug encapsulation. Through hydrogen bonding with the PEG moiety of the amphiphilic PEG-*b*-AGE coating, PEG550 may provide a protection further stabilizing CAPE molecules encapsulated in the hydrophobic layer of the coating polymer (Xue et al., 2022). The loading efficiency of CAPE after stabilized by PEG550 was measured to be 48.7% ± 3.3% by UV-Vis spectroscopy. Notably, the introduction of PEG550 did not result in an obvious change in the D_H_ of IONP/CAPE (Figure 2C), suggesting molecules of CAPE and PEG550 were both encapsulated in the coating layer of IONP. The zeta-potential for PEG-*b*-AGE coated IONP was measured at 3.32 ± 1.21 mV, consistent with previously reported results (Li et al., 2015). After encapsulation, a zeta-potential of 1.75 ± 1.03 mV was found for IONP/CAPE, similar to that of IONP with no statistical significance (*p* > 0.05). The results suggested a minimal effect of CAPE encapsulation on the surface charge of IONP. The functionalization of RGD as targeting ligands, on the other hand, increased the D_H_ of RGD-IONP/CAPE to 37.1 nm with the PDI of 0.14 (Figure 2C), which is also consistent with the results reported previously (Li et al., 2015). The successful conjugation of RGD was further validated by the change of zeta-potentials of IONP formulations. IONP/CAPE exhibited a slightly positive surface charge due to the presence of –NH_2_ groups from PEG-*b*-AGE polymer on the surface. After conjugation with RGD, a negative zeta-potential of −11.33 ± 2.21 mV was found for RGD-IONP/CAPE, which can be ascribed to the replacement of –NH_2_ groups with RGD molecules bearing negatively charged –COO^-^ groups. The number of RGD molecules conjugated to each IONP was found to be 78 ± 24, after measuring using a microBCA assay. The stability of CAPE loading was investigated by monitoring the release of CAPE from RGD-IONP/CAPE in buffers mimicking lysosomal (pH 5.5), physiological (pH 7.4) and routine fluorescence labeling conditions (pH 9.5), respectively. It was noticed that CAPE loading was stable at pH 7.4 and 9.5 with <3% changes over 48 h, whereas approximately 80% of CAPE were released within 48 h at pH 5.5 (Figure 2D). These results suggested that RGD-IONP/CAPE was stable during modifications under neutral or basic conditions, and could be applicable for drug release triggered by an acidic environment, such as the tumor and lysosomes of cancer cells after delivery. The stability of RGD-IONP/CAPE was also monitored for 7 days for the change of hydrodynamic size and CAPE loading (Figure 2E). The results indicated no statistically significant change in the hydrodynamic size of RGD-IONP/CAPE, which varied from 27.90 ± 6.83 nm (day 1) to 29.67 ± 8.09 nm (day 7) with an increase of ∼6.0%. Similarly, the loading efficiency of CAPE changed from 48.7% ± 3.3% (day 1) to 46.6% ± 2.5% (day 7), with a decrease of ∼4.3% but not statistically significant. With the validated stability, the MRI contrast capability of RGD-IONP/CAPE was then evaluated for future theranostic applications *in vivo*. To examine whether the transverse T_2_-weighted MRI contrast enhancement of IONP were affected by the encapsulation of CAPE and surface functionalization with RGD, solutions of IONP and RGD-IONP/CAPE were prepared, respectively, with the Fe concentrations of 0.03, 0.06, 0.12, and 0.25 mM. The solutions of IONP and RGD-IONP/CAPE demonstrated similar T_2_-weighted MRI contrast enhancement, and the transverse relaxivity (r_2_) was found to be 58.21 mM^-1^S^−1^ for IONP and 54.19 mM^-1^S^−1^ for RGD-IONP/CAPE, measured by a multi-TE sequence (Figure 2F), suggesting a negligible effect of CAPE encapsulation and surface modification of RGD on the IONP for MRI contrast enhancement.

### Targeting specificity of RGD-IONP/CAPE for MM cells

**FIGURE 3:**
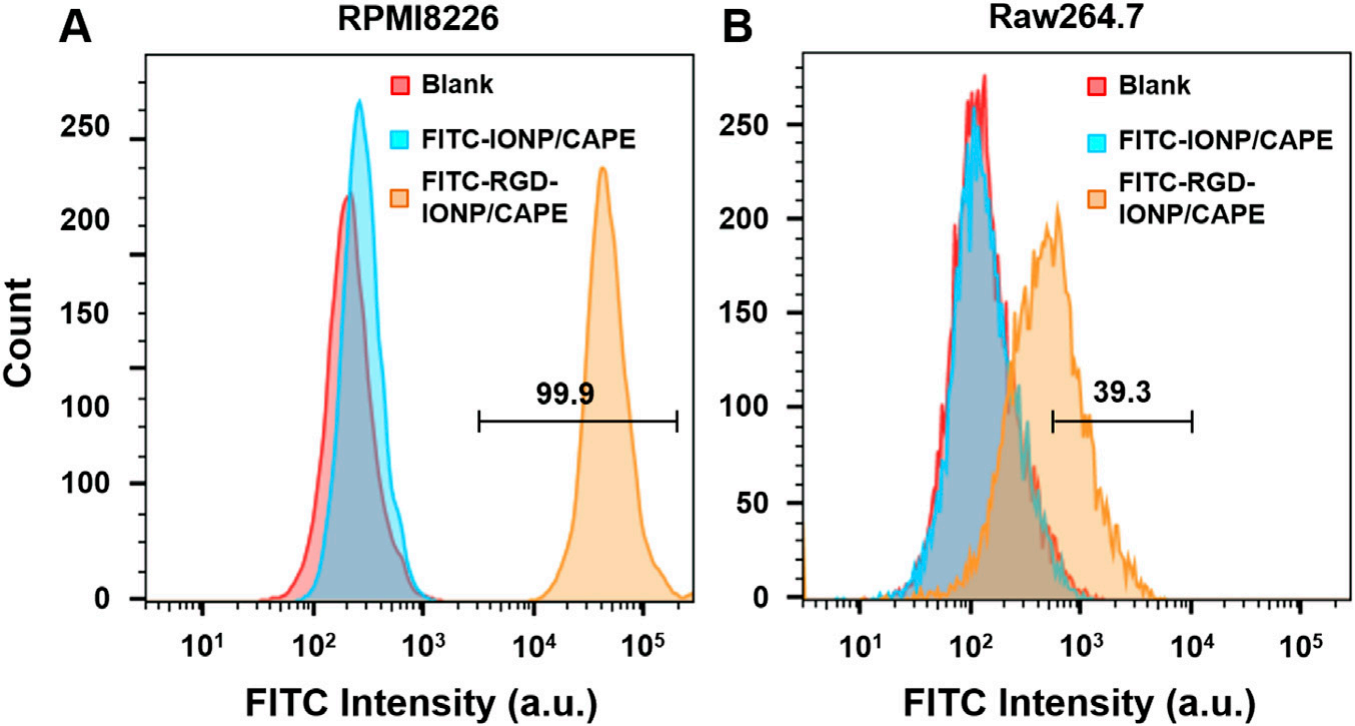
Cellular uptake of FITC-labeled non-targeted IONP/CAPE and α_v_β_3_ integrin-targeted RGD-IONP/CAPE by human MM RPMI8826 cells **(A)** and mouse macrophage RAW264.7 cells **(B)** analyzed by flow cytometry.

We validated the specificity of RGD-IONP/CAPE for targeting α_v_β_3_ integrin on RPMI8826 human MM cells using FACS. RAW264.7 mouse macrophage cells were used as the negative control due to the low expression level of α_v_β_3_ integrin (Antonov et al., 2004) as well as its phagocytic nature resembling the immune cells responsible for clearing exogenous substances *in vivo* (Lavin et al., 2015). With FITC fluorescence labeling, RPMI8826 cells treated with non-targeted IONP/CAPE showed a mostly overlapped histogram with that of non-treated RPMI8826 cells, while those treated with RGD-IONP/CAPE demonstrated a distinct population from the no treatment control (Figure 3A). The results suggested that little IONP/CAPE was taken up by the MM cancer cells, whereas α_v_β_3_ integrin-targeted RGD-IONP/CAPE was bound or taken up by the majority (99.9%) of cancer cells. Similarly, RAW264.7 macrophages showed little uptake of non-targeted IONP/CAPE, as shown by the highly overlapped histograms from no treatment control and FITC-IONP/CAPE treatment group. The flow cytometry analysis for macrophages treated with FITC-RGD-IONP/CAPE revealed a shift in the histogram, suggesting 39.3% of macrophages were labeled with FITC-RGD-IONP/CAPE (Figure 3B). Worth noting, the FITC intensity measured from RPMI8826 MM cells was approximately 100 times stronger than RAW264.7 macrophages after treating with FITC-RGD-IONP/CAPE, indicating the much higher cellular uptake of the nano-complex by the MM cells. Taking together, the RGD-IONP/CAPE exhibited a cellular uptake highly dependent on the α_v_β_3_ integrin expression levels.

### Cytotoxicity of RGD-IONP/CAPE in MM cells

**FIGURE 4:**
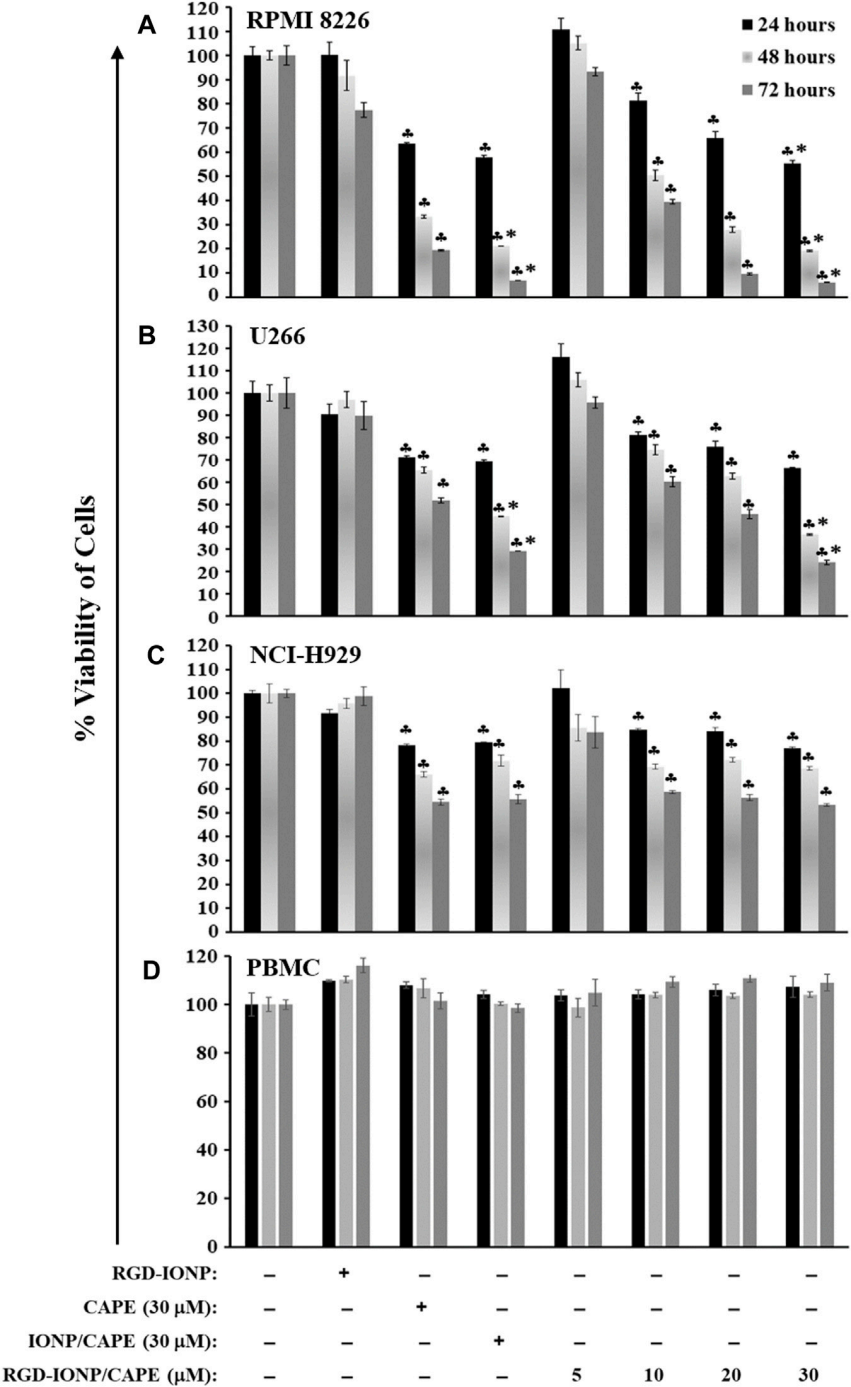
The cytotoxic effects of RGD-IONP/CAPE in human RPMI8226 cells **(A)**, U266 cells **(B)**, NCI-H929 cells **(C)**, and human normal PBMCs **(D)**. ♣: *p* < 0.05 *versus* cell only control. *: *p* < 0.05 *versus* CAPE (30 μM) only control. The data are presented as mean ± SD of three independent replicates.

We previously reported that CAPE inhibited the growth of MM cells in both dose- and time-dependent manners (Marin et al., 2019). In the present study, we evaluated the effect of IONP-carrier-loaded CAPE on the growth of three human MM cell lines, including RPMI8226, NCI-H929 and U266. These MM cells were treated with RGD-IONP/CAPE at different CAPE concentrations (0, 5, 10, 20 and 30 μM), CAPE alone (30 μM), non-targeted IONP/CAPE (30 μM), and RGD-IONP vehicle control. Viabilities of treated MM cells were examined after 24, 48 and 72 h, respectively. The data showed that RGD-IONP/CAPE inhibited the growth of all three types of human myeloma cells, leading to dose- and time-dependent decrease of cell viabilities (Figure 4). The inhibitory effect of RGD-IONP/CAPE begins with 10 μM after 24-h incubation in all MM cell lines tested. In RPMI8226 cells, the cell viability dropped to 55%, 19%, and 6% after 24, 48, and 72-h incubation of RGD-IONP/CAPE, respectively. Similarly, 66%, 37%, and 24% decrease for U266 cells, and 77%, 69%, and 53% for NCI-H929 cells were observed after 24, 48, and 72-h incubation, respectively. We then determined the IC50 of RGD-IONP/CAPE for the 3 MM cell lines by incubating RGD-IONP/CAPE with the cancer cells for 72 h (Figure 4). The IC50 was found to be 7.97 ± 1.39 μM for RGD-IONP/CAPE in RPMI8226 cells, which is about 50% less than that of CAPE (15.91 ± 1.10 μM) (Marin et al., 2019). Similarly, the IC50 of 16.75 ± 1.62 μM in U266 cells and 24.38 ± 1.71 μM in NCI-H929 cells were determined for RGD-IONP/CAPE, lower than those of CAPE alone (55.79 ± 7.91 μM in U266 cells; 30.66 ± 1.61 μM in NCI-H929 cells). Meanwhile, RGD-IONP/CAPE, CAPE alone, and non-targeted IONP/CAPE did not affect the viability of PBMCs used as normal human cell control (Figure 4D). Taken together, these results suggested that RGD-IONP carriers not only render a fully water-soluble approach for using CAPE with an enhanced efficacy, but also allow for the MM-cell-specific cytotoxicity with biocompatibility for normal blood cells.

### Apoptosis of RPMI8226 MM cells induced by RGD-IONP/CAPE

**FIGURE 5:**
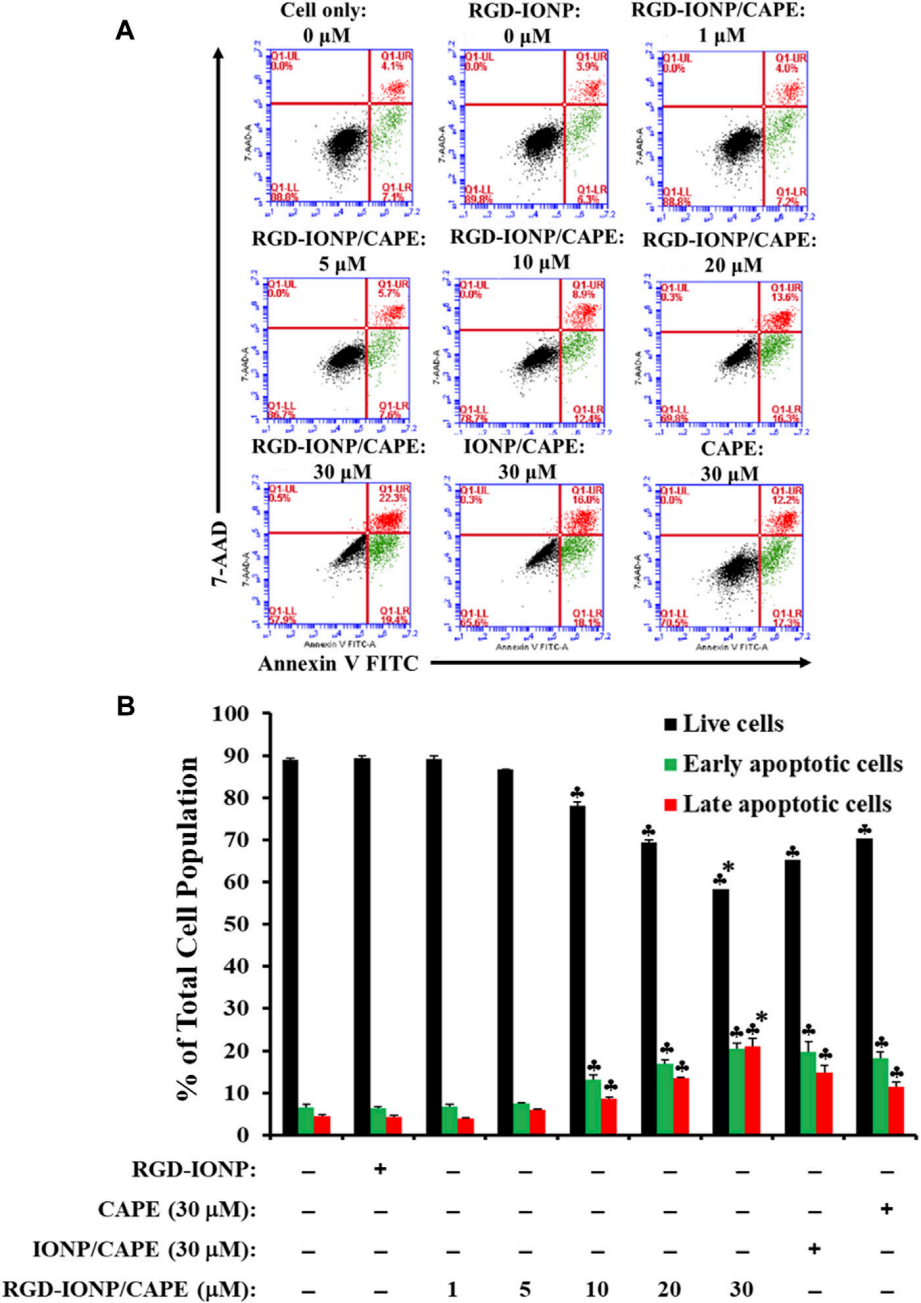
Apoptosis of RPMI8226 cells induced by RGD-IONP/CAPE within 24 h. Annexin V FITC vs. 7-AAD plots from the gated cells show the populations corresponding to viable and nonapoptotic (Annexin V^−^ 7-AAD^−^, black dots), early (Annexin V^+^ 7-AAD^–^, green dots), and late (Annexin V^+^ 7-AAD^+^, red dots) apoptotic cells **(A)**. Percentages of live and apoptotic cells from each treatment group obtained from flow cytometry analysis are compared **(B)**. ♣: *p* < 0.05 *versus* cell only control. *: *p* < 0.05 *versus* CAPE (30 μM) only control. The data are presented as mean ± SD of three independent experiments.

To examine whether the cytotoxicity of RGD-IONP/CAPE in MM cells is due to the same mechanism of apoptosis as we discovered previously (Marin et al., 2019), using RPMI8226 cells as an example, we first performed a flow cytometry analysis on RPMI8226 cells treated with RGD-IONP/CAPE (at CAPE concentrations ranging from 1 to 30 µM), CAPE alone (30 µM), non-targeted IONP/CAPE (30 µM CAPE), and vehicle control RGD-IONP (at the equivalent Fe dosage to 30 µM RDG-IONP/CAPE) for 24 h followed by double staining of the cells using Annexin-V FITC and 7-AAD (Figure 5A). The results revealed a dose-dependent effect of RGD-IONP/CAPE in inducing cancer cell apoptosis. The early (Annexin-V^+^/7-AAD^-^) and late (Annexin-V^+^/7-AAD^+^) apoptotic cell populations of RPMI8226 cells increased from 6.8% ± 0.6% to 20% ± 1.4% and 4.0% ± 0.1% to 21% ± 1.8%, respectively, when the doses increased from 1 to 30 μM (Figure 5B). In addition, the delivery of CAPE to MM cells was significantly (*p* < 0.0001) improved when RGD-IONP were used as carriers, evidenced by the further reduced populations of non-apoptotic RPMI8226 cells after a 24-h treatment with RGD-IONP/CAPE (58% ± 0.5%) *versus* those of non-targeted IONP/CAPE (65% ± 0.5%) and CAPE alone (70% ± 0.3%) at the CAPE concentration of 30 μM (Figure 5B). It is noticeable that the population of late apoptotic cells was significantly (*p* < 0.0001) larger in the group of RGD-IONP/CAPE (21% ± 1.8%) than non-targeted IONP/CAPE (15% ± 1.8%) and CAPE alone (11% ± 1.1%) at the same CAPE dosage (30 μM). The populations of early apoptotic cells, however, were similar among RGD-IONP/CAPE (20% ± 1.4%), non-targeted IONP/CAPE (20% ± 2.3%), and CAPE alone (18% ± 1.4%). On the contract, the vehicle control RGD-IONP showed no difference in live or apoptotic cell population comparing to the cell only control.

**FIGURE 6:**
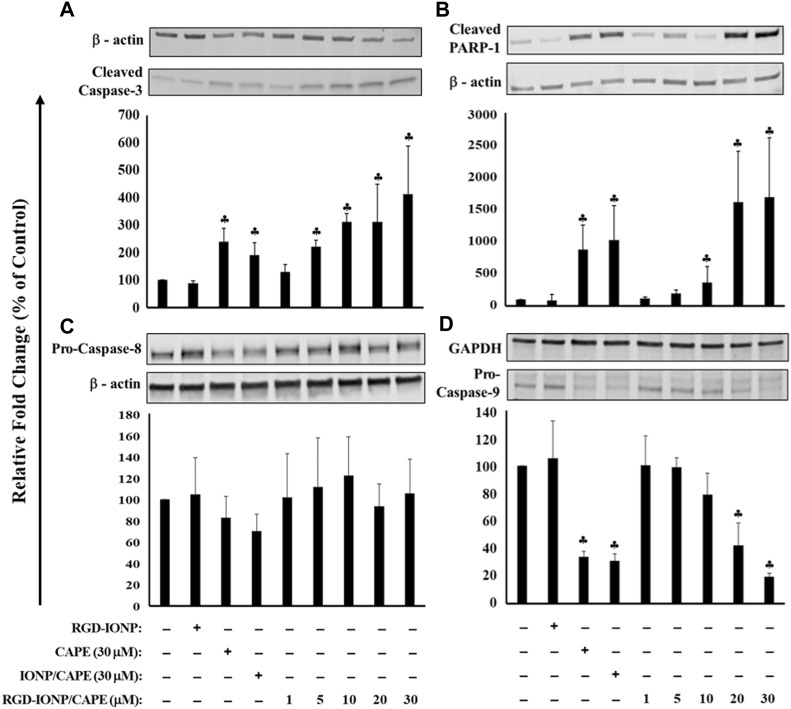
Apoptosis of RPMI8226 cells induced by 24h treatment of RGD-IONP/CAPE through caspase-3 mediated intrinsic apoptotic pathway. This is evidenced by a dose-dependent increased protein expression of cleaved caspase-3 **(A)** and its downstream target PARP-1 **(B)**, and decreased protein expression of pro-caspase-9 **(C)** with no significant change of pro-caspase-8 level **(D)**. Parent CAPE and IONP/CAPE exhibit similar trend as RGD-IONP/CAPE but to a less extent. ♣: *p* < 0.05 *versus* cell only control. The data are presented as mean ± SD of three independent experiments.

To further explore the apoptotic mechanism, we examined caspase-3, a key player in apoptosis, and poly (ADP-ribose)polymerase-1 (PARP-1), the caspase-3 downstream target involved in repair of DNA damage (Thapa et al., 2021). Following a 24-h treatment of RGD-IONP/CAPE, cleavage of caspase-3 and PARP-1 were observed as shown in Figures 6A,B. Expression of caspase-3 in active form was significantly increased in a dose-dependent manner (Figure 6A). Similarly, expression of PARP-1 in cleaved form was significantly elevated with increased doses (Figure 6B). When comparing the extent of caspase-3 and PARP-1 activation, we observed higher fold changes in caspase-3 and PARP-1 protein expression in the group of RGD-IONP/CAPE than that of non-targeted IONP/CAPE and CAPE alone at the same CAPE dosage of 30 μM (Figures 6A,B). Because caspase-3 is a downstream effector from both intrinsic and extrinsic apoptotic pathways, which are initiated by caspase-9 and caspase-8, respectively (McComb et al., 2019), we then evaluated the expression of caspase-8 and 9 in RPMI8226 cells. Expression of caspase-9 in pro-form was found significantly decreased, while that of caspase-8 in pro-form was not under the same condition (Figures 6C,D). Taken together, these results suggested that RGD-IONP/CAPE induces apoptosis of RPMI8226 cells through caspase-3-mediated intrinsic apoptotic pathway.

### Apoptosis induction in a TW model

**FIGURE 7:**
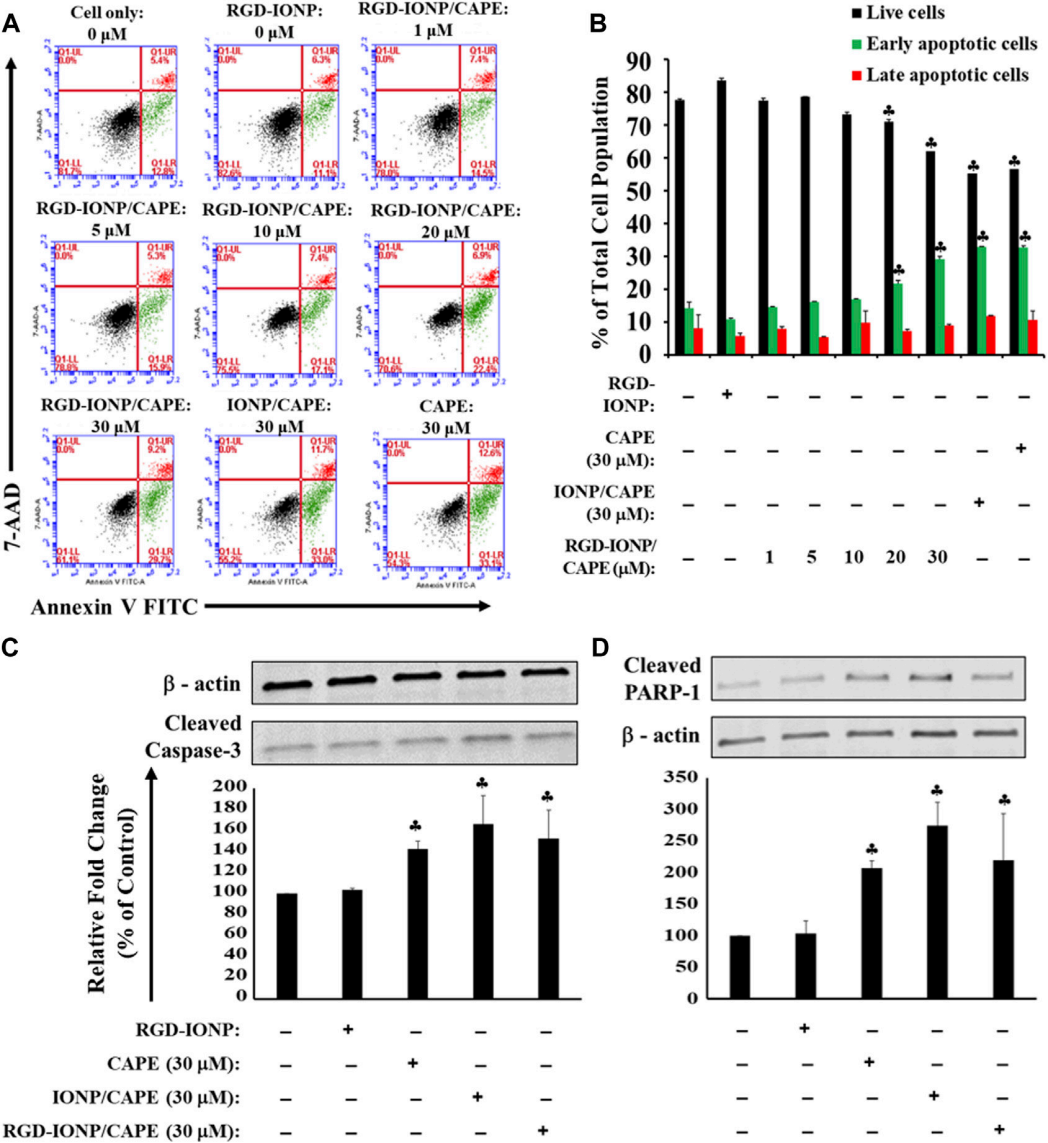
Apoptosis of RPMI8226 cells induced by RGD-IONP/CAPE within 24 h in the TW model. Dose-dependent induction of RPMI8226 apoptosis was observed in the presence of HS-5 cells **(A)**. Percentages of live and apoptotic cells from each treatment group are compared **(B)**. No significant difference in apoptotic cells is observed among parental CAPE, IONP/CAPE, and RGD-IONP/CAPE though. This is also evidenced by a similar level of increased protein expression of cleaved caspase-3 **(C)** and its downstream target PARP-1 **(D)** among these three groups examined. ♣: *p* < 0.05 *versus* cell only control. The data are presented as mean ± SD of three independent experiments.

MM cells are homed within the bone marrow microenvironment, which protects the MM cells from easily accessing by anti-MM drugs (Manier et al., 2012), supports the proliferation and survival of MM cells, and develops resistance of MM cells against chemotherapeutic drugs (Noll et al., 2014; Ria and Vacca, 2020). The microenvironment in bone marrow features a complex network consisting of cells such as stromal cells and immune cells, signaling molecules cytokines and growth factors, and extracellular matrix proteins (Kawano et al., 2015). Hence, we adopted a previously used TW model by co-culturing human RPMI8226 cells and HS-5 stromal cells to mimic the bone marrow microenvironment (Perez et al., 2008). After treating the cells with RGD-IONP/CAPE for 24 h, a dose-dependent apoptosis was observed for RPMI8226 cells in the presence of HS-5 stromal cells, with the viable cell population decreased from 84% ± 1.3% to 62% ± 1.3% when the CAPE dosages increased from 0 to 30 μM (Figure 7A). However, no significant difference in apoptotic population was observed for RPMI8226 cells when treating with RGD-IONP/CAPE, non-target IONP/CAPE or CAPE alone at the same CAPE dosage. To validate whether death of MM cells still stemmed from the apoptosis induced by RGD-IONP/CAPE, we examined the expression of cleaved caspase-3 and PARP-1 for RPMI8226 cells in the TW model. The results showed the expressions of cleaved caspase-3 and PARP-1 of RGD-IONP/CAPE treated cells were significantly increased, comparing to the vehicle control RGD-IONP. However, difference in expression levels among the groups of RGD-IONP/CAPE, non-target IONP/CAPE and CAPE alone were not significant for neither cleaved caspase-3 nor PARP-1. The activation of apoptosis-related factors correlates well with the elevation of apoptotic cell population after treatment groups of RGD-IONP/CAPE, non-target IONP/CAPE and CAPE alone, confirming the induction of apoptosis in the TW model. Taken together, these data suggested that caspase-3 mediated apoptosis of RPMI8226 cells was induced by RGD-IONP/CAPE in the TW model with the presence of HS-5 stromal cells, however, with an attenuated efficacy for RGD-IONP/CAPE. The results also indicated investigations for the microenvironment of MM in the bone marrow, such as signal pathways between MM and stromal cells, will be necessary to enhance the effectiveness or efficacies of chemotherapies and other treatment strategies for MM.

## Discussion

In spite of the doubled 5-year overall survival rate for MM to above 50% over the past decade (Daniele et al., 2022), as our understanding for the disease biology advances, the treatment strategies for MM have been, and will keep, evolving with persisting demands for more appropriate therapeutics. As an example, current treatment options, such as the proteasome inhibitor bortezomib and immunomodulatory agent lenalidomide, are subject to development of drug resistance in almost all patients (Zhang et al., 2020). Several previous *in vitro* studies by us and others have suggested the different mechanisms of CAPE from current chemotherapy agents for MM, making CAPE a promising alternative. However, as a lipophilic small molecule compound with an alkyl caffeate ester, CAPE is subject to distribution and deep penetration in healthy organs and esterase-initiated hydrolysis *in vivo* (Celli et al., 2007; Wang et al., 2007; Wang et al., 2009).

As an effective strategy to improve the delivery of drugs, utilizing nanomaterials as carriers to increase the bioavailability of CAPE has been reported by several laboratories (Lee et al., 2015; Wang et al., 2020a; Colpan and Erdemir, 2021). Polymeric and lipid nanoparticles are the most investigated nano-carriers for CAPE due to high loading efficiency, ease of preparation and high lipophilicity of CAPE. However, evaluations of these systems *in vivo* have been sporadic. Drawbacks of polymeric and lipid nanoparticle carriers may include the relatively large diameters (several hundred nm) affecting the effective NP delivery to tumors along with limited surface modification methods for conjugating tumor targeting ligands. Hence, we investigated the application of an anti-biofouling IONP to facilitate the delivery of CAPE. As a result, CAPE can be successfully encapsulated into the IONP. The loading can be stabilized by the addition of PEG550 to form an extra protecting outer layer likely through hydrogen bonding between PEG550 and the PEG moieties of coating polymer. The nano-formulation demonstrated a stability in physiological and basic conditions, and triggered release of CAPE in acidic environment. Such feature potentially allows the nano-formulation to stably carry CAPE during blood circulation and specifically release the drug payload in the tumor environment or lysosomes of cancer cells after receptor-mediated internalization. The anti-biofouling IONP carriers have shown reduced non-specific interactions with the biological environment *in vivo*, such as the non-specific uptake by macrophages in the blood. The carrier holds potential in avoiding fast clearance by immune cells *in vivo* and enhance the targeted delivery of therapeutics to cancer cells after functionalized with targeting ligands. In addition, the developed RGD-IONP/CAPE nano-formulation can be used for image-guided drug delivery by T_2_-weighted MRI, as other IONP formulations (Huang et al., 2016; Dadfar et al., 2019).

The anti-myeloma potential of CAPE was investigated previously, confirming its inhibitory effect and induction of apoptosis in human MM cells (Altayli et al., 2015). In addition to the induction of oxidative stress (Marin et al., 2019), this compound was reported to downregulate the expression of IRF-4, Sp-1, and the IKZF-1-IRF4-MYC axis, key factors for myeloma cell growth and survival, in MM cells (Murugesan et al., 2020). Here, for the first time to our knowledge, we applied IONP with the conjugation of MM-cell-targeted ligand (RGD) to deliver CAPE, and examined its cytotoxic and apoptogenic effects. Evaluating the inhibitory effect of RGD-IONP/CAPE on the growth of MM cells *in vitro* is the first step in exploring the potential of this therapeutic strategy against deadly MM. Our results showed, in addition to the dose and time-dependent cytotoxicity (Figure 4), RGD-IONP/CAPE is more potent than CAPE alone when comparing the respective IC50 values in the 3 MM cell lines tested. Importantly, the cytotoxic effect of RGD-IONP/CAPE was confirmed to be specific to MM cell, as no cytotoxicity for the normal human PBMCs was detected. It was also reported that CAPE did not affect the viability of primary PBMCs isolated from healthy volunteers at a dose of up to 50 μg/ml (176 μM) (Cavaliere et al., 2009). Our data confirmed the non-toxicity of CAPE in PBMCs. The CAPE-loaded NPs showed no significant difference in altering the cell viability compared to CAPE (Figure 4D). The observed death of MM cells was later found through the apoptosis induced by RGD-IONP/CAPE, which led to more apoptotic cells at later stage than that of CAPE alone (Figure 5). Interestingly, as revealed by the data, the population of late, not early, apoptotic cells is significantly increased after the treatment of RGD-IONP/CAPE (21% ± 1.8%) comparing to non-targeted IONP/CAPE (15% ± 1.8%) and CAPE alone (11% ± 1.1%). Although CAPE was reported to induce apoptosis (Altayli et al., 2015; Murugesan et al., 2020), it is the first time, to our best knowledge, to observe the differential response of early and late apoptosis, due to the application of RGD-IONP. In addition, RGD-IONP/CAPE induced apoptosis is mediated through the same caspase-3 pathway as CAPE. Further mechanistic investigation reveals that caspase-9, not caspase-8, is responsible for the upstream activation of this caspase-3 mediated apoptosis pathway.

Because bone marrow microenvironment, especially the interaction between bone marrow stromal and MM cancer cells, plays a protective role in the disease progression and development of resistance to drugs for MM (Ria and Vacca, 2020; Garcia-Ortiz et al., 2021), we further evaluated the cytotoxic effects of RGD-IONP/CAPE in a TW model mimicking the MM microenvironment *in vivo*. Interestingly, RGD-IONP carriers did not render CAPE a greater apoptogenic effect for MM cells in the TW model based on the flow cytometry data (Figure 7B). As the inductions of caspase-3 mediated apoptotic pathway through the treatment of CAPE, IONP/CAPE, and RGD-IONP/CAPE were confirmed by the increased expression of caspase-3 and PARP-1, it is reasonably to hypothesize that the interactions between HS-5 bone marrow stromal cells and RPMI8226 MM cells provided a microenvironment to “buffer” the cytotoxicity from CAPE, IONP/CAPE and RGD-IONP/CAPE. The attenuated effectiveness of RGD-IONP/CAPE highlights the importance of taking tumor microenvironment into consideration while evaluating the efficacy of a treatment. Further studies will focus on strategies to disrupt the protecting microenvironment of bone marrow for MM cells to exert the efficacy of treatment, such as the developed RGD-IONP/CAPE. The incorporation of RGD-IONP as carriers for delivery of CAPE *in vivo* is important, as they hold potentials in navigating hydrophobic CAPE molecules through the complex biological environment and tumor microenvironment to the targeted site. Our future efforts will also include the application of more potent delivery systems, such as ultrafine IONP with better tumor accumulation and penetration (Li et al., 2022), development of more MM microenvironment recapitulating models through 3D bioprinting technique (Wu et al., 2022), and the synergistic effects of CAPE in combination with current treatment options such as bortezomib and lenalidomide.

## Conclusion

In this study, we prepared a CAPE-based nano-formulation (RGD-IONP/CAPE) for treating MM, by encapsulating the potent yet hydrophobic CAPE molecules to anti-biofouling PEG-*b*-AGE polymer coated IONP (IONP/CAPE), followed with conjugation of α_v_β_3_-integrin targeted RGD peptides. We characterized the colloidal stability, CAPE loading and release profiles, targeting specificity and cytotoxicity for MM cells. As a conclusion, the developed nano-formulation offers an approach to apply CAPE in a more biocompatible way with a greater efficacy than the free drug. More importantly, the nano-formulation allows for specific deliveries and subsequent cytotoxicity for MM cells through a caspase-3 mediated apoptosis. This study has laid the foundation for targeted delivery of CAPE to treat MM and has provided justification for further evaluation of this natural compound as a potential anti-myeloma agent or in combination treatment with current chemotherapeutics.

## Data Availability

The raw data supporting the conclusion of this article will be made available by the authors, without undue reservation.
