# Supplementary figures and images for: Tumor integrin targeted theranostic iron oxide nanoparticles for delivery of caffeic acid phenethyl ester: preparation, characterization, and anti-myeloma activities
Source: Front Pharmacol. 2024 Mar 6;15:1325196. doi: 10.3389/fphar.2024.1325196 (PMC10952826; doi:10.3389/fphar.2024.1325196)

**Figure S1:**

**
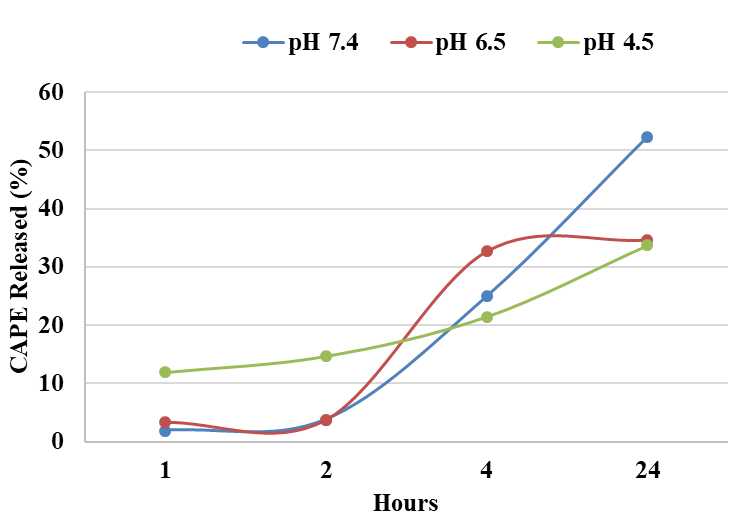
**

Supplement: Supplementary file 1 [file Table1.DOCX]
